# Adaptive evolution of Toll-like receptor 5 in domesticated mammals

**DOI:** 10.1186/1471-2148-12-122

**Published:** 2012-07-24

**Authors:** Sarah A Smith, Oliver C Jann, David Haig, George C Russell, Dirk Werling, Elizabeth J Glass, Richard D Emes

**Affiliations:** 1 School of Veterinary Medicine and Science, University of Nottingham, Sutton Bonington LE12 5RD, UK; 2 The Roslin Institute and Royal Dick School of Veterinary Studies, The University of Edinburgh, Easter Bush, Midlothian, EH25 9RG, UK; 3 Royal Veterinary College, University of London, Hawkshead Lane, Hatfield, Herts, AL9 7TA, UK; 4 Moredun Research Institute, Pentlands Science Park, Bush Loan, Penicuik, Midlothian, EH26 0PZ, UK

**Keywords:** Toll-like receptor, SNP, Adaptive evolution, Positive selection, Sheep, Cattle

## Abstract

**Background:**

Previous studies have proposed that mammalian toll like receptors (TLRs) have evolved under diversifying selection due to their role in pathogen detection. To determine if this is the case, we examined the extent of adaptive evolution in the TLR5 gene in both individual species and defined clades of the mammalia.

**Results:**

In support of previous studies, we find evidence of adaptive evolution of mammalian TLR5. However, we also show that TLR5 genes of domestic livestock have a concentration of single nucleotide polymorphisms suggesting a specific signature of adaptation. Using codon models of evolution we have identified a concentration of rapidly evolving codons within the TLR5 extracellular domain a site of interaction between host and the bacterial surface protein flagellin.

**Conclusions:**

The results suggest that interactions between pathogen and host may be driving adaptive change in TLR5 by competition between species. In support of this, we have identified single nucleotide polymorphisms (SNP) in sheep and cattle TLR5 genes that are co-localised and co-incident with the predicted adaptive codons suggesting that adaptation in this region of the TLR5 gene is on-going in domestic species.

## Background

Toll-like receptors (TLRs) are type 1 transmembrane glycoproteins expressed on the cell surface and intracellular compartments of many cell types including epithelial cells and a variety of immune cells such as macrophages and dendritic cells. TLR ligands include pathogen-associated molecular pattern (PAMP) molecules, and TLRs are amongst the first receptors to respond to pathogen presence [1], hence their key role in innate immunity to infection. At least 10 TLRs have been identified in mammals and collectively these recognize a wide repertoire of microbial organisms and pathogens including bacteria, viruses, protozoa and fungi [2]. The TLR protein is comprised of three main regions: an extracellular pattern-recognition receptor domain (ECD), a transmembrane region and an intracellular TIR signalling domain [3]. The signalling domain is highly conserved across the TLRs. In contrast, the ECD involved in pathogen detection is often variable [2].

TLR5 is known to bind bacterial flagellin [4]. Both flagellin (e.g. of *E.coli*) [4]), and the ECD of TLR5 in primates [5] and other mammals [6] show evidence of adaptive positive selection. This suggests that interspecies competition between host and pathogen is likely to be driving the co-evolution of pathogen and host. In support of this, species-specific single nucleotide variations in the TLR5 gene exist and a single nucleotide polymorphism (SNP) in the ECD of mouse, chicken and human TLR5 is associated with a species-specific response to flagellin [7,8].

The domestication of livestock by selection of desirable traits gave rise to the concept of breeds over 200 years ago [9]. This formation of breeds by selective interbreeding offers a unique opportunity to examine an accelerated process of natural selection. To investigate the evolution of the TLR5 gene in domestic livestock compared to other mammals we used phylogenetic methods to identify species-specific and branch-specific evidence of positive selection. To investigate the potential role of recent variation on evolution of the TLR5 gene we also identified known and novel SNPs in the coding region of TLR5 of sheep and cattle breeds.

## Results

### Evidence for adaptive evolution in mammalian TLR5

Positive diversifying selection acting on a gene can be inferred when the ratio of non-synonymous (*d*_*N*_) to synonymous (*d*_*S*_) substitution rates is greater than 1. This ratio *d*_*N*_/*d*_*S*_ (also known as omega) provides a method to compare the evolutionary history of codons and lineages [10]. The parameters *d*_*N*_ and *d*_*S*_ can be estimated by a number of approaches. We applied the codon models of PAML [11] to infer estimates of parameters under a maximum likelihood framework. The results are summarized in Table 1 and Table 2. Complete results and parameter estimates for all PAML analyses are given as Additional file 1 and Additional file 2. 

**Table 1:** Detection of positive selection across mammalian TLR5

**Analysis**	***LRT M1a versus M2a (significance)***	***LRT M7 versus M8 (significance)***	***d***_***N***_***/dS***_**s**_**(model)**	**Sites under positive selection**^**a**^
All Mammals	18.87 (p < 0.001)	28.61 (p < 0.001)	2.85 (M2), 1.56 (M8)	G104*
				H592*
				A659*

Results of PAML site specific analysis of positive selection across mammalian TLR5 and branch-site analysis. Positively selected codons detected by both M2a and M8 models are shown * = P*b* > 95%.

**Table 2:** Detection of positive selection of mammalian TLR5 using the branch-sites test

**Foreground branch(es)**	**LRT Foreground*d***_***N***_***/d***_***S***_ **= 1 versus foreground*d***_***N***_***/d***_***S***_ **> 1**	***d***_***N***_***/d***_***S***_**(branch)**	**Sites under positive selection**^**a**^
Artiodactyls	35.61 (p < 0.001)	4.76 (foreground)	L34*
			S268**
			I295*
			I307*
			I393*
			Q495*
			I621*
			E630**
Cow	21.84 (p < 0.001)	140.91 ( foreground )	I393**
			Q495*
			H720*
Sheep	18.10 (p < 0.01)	493.28 ( foreground )	K326*
Pig	11.89 (p < 0.05)	29.07 (foreground )	E630**

Results of branch-sites analysis of positive selection within mammalian TLR5. ^a^ posterior probabilities (P*b*) of codons existing in *d*_*N*_*/d*_*S*_ > 1 site class * = P*b* > 0.95 and ** P*b* > 0.99.

When comparing rates of codon evolution in TLR5 since the divergence of the mammals (sites analysis, see methods) significant evidence (posterior probability P_*b*_ > 0.95) was found to suggest that positive selection has been acting on three codons (G104, H592, A659) Table 1. When using the branch-site test (see methods) to compare each lineage independently, significant evidence was obtained that the sloth, sheep, cattle and pig lineages were each evolving at an elevated rate compared to other branches of the phylogeny. Three positively-selected codons (I393, Q495, H720, P_*b*_ > 0.95) were detected in the cattle lineage. One codon (K326, P_*b*_ > 0.99) was identified in the sheep lineage and one codon (E630, P_*b*_ > 0.95) was identified in the pig lineage (Table 2). These sites are in addition to those identified as having evolved under positive selection across the mammalian lineage.

**Figure 1:**
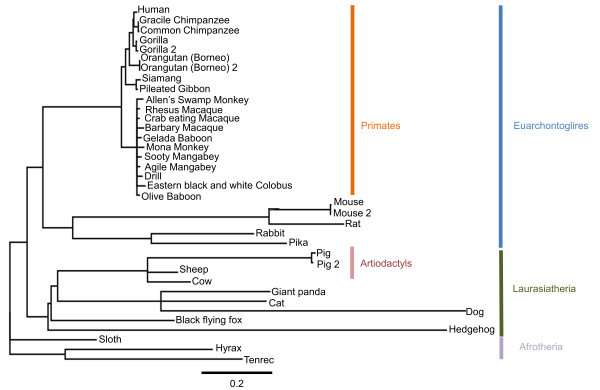
**Phylogeny of species analysed.** Generalised phylogeny of the species and clades analysed. The topology of the tree is based on an accepted mammalian phylogeny [32]. Branch lengths in substitutions per codon are calculated under the M0 model of PAML [11].

Using the multiple branch-sites analysis (see methods), positive selection of TLR5 was detected in the artiodactyls, which contain the domestic ungulate species (cow, sheep and pig), but not in the laurasiatheria (of which artiodactyls are a component clade) or in the separate euarchontoglires and primate clades (Figure 1). In the artiodactyls, eight codons of TLR5 were detected with significant evidence of positive selection in (L34, S268, I295, I307, I393, Q495, I621, E630, (P_*b*_ > 0.95). Five codons (L34, S268, I295, I307, I621) were only identified as having evolved under positive selection when the members of the artiodactyla were combined in the multiple branch-sites analysis. Three codons (I393, Q495, E630) were detected previously in ungulate species-specific lineages (Table 2).

**Figure 2:**
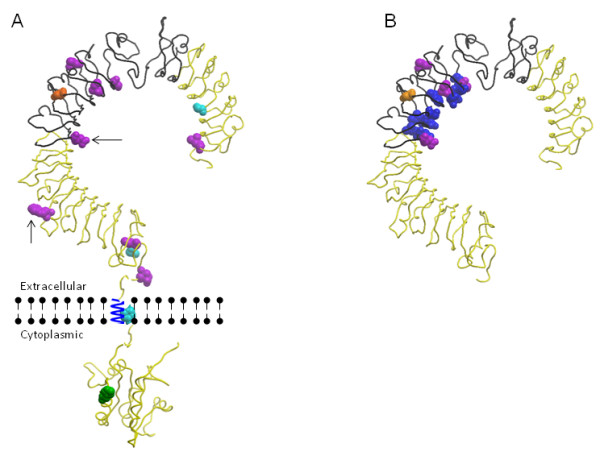
**Localisation of TLR5 sites of positive selection.A**) predicted tertiary protein structure of bovine TLR5 and sites of positive selection. **B**) predicted tertiary protein structure of ECD and sites of positive selection and conserved amino acids within putative flagellin binding region. Black backbone depicts putative flagellin binding region; Light blue amino acids = Positive selection acting on all mammals (G104, H592, A659); Purple amino acids = Positive selection acting on domestic ungulates (L34, S268, I295, I307, I393, Q495, I621, E630); Orange amino acid = positive selection in sheep lineage (K326), Green amino acids and black arrows = positive selection in cattle lineage (I393,Q495,H720 ); Dark blue amino acids = Conserved amino acids predicted to be involved in the detection of flagellin.

When mapped onto the predicted tertiary structure of bovine TLR5 (PDB 2a0zA [12] and 1fyv [13]) the location of sites of positive selection detected by all approaches revealed a bias in their distribution. Eleven (L34, G104, S268, I295, I307, K326, I393, Q495, H592, I621, E630) of the thirteen positively-selected codons encode amino acids in the ECD (Figure 2a). When the total number of sites in the ECD compared to the rest of the protein are accounted for, this enrichment remains statistically-significant (Fisher Exact Test: *P* = 0.03). Additionally five of these (S268, I295, I307, K326, I393) which exhibited evidence of positive selection in the artiodactyl clade are located within the putative flagellin-binding region of the ECD close to the conserved concave surface-associated with ligand binding [7] (Figure 2b). 

### SNP Detection

**Figure 3:**
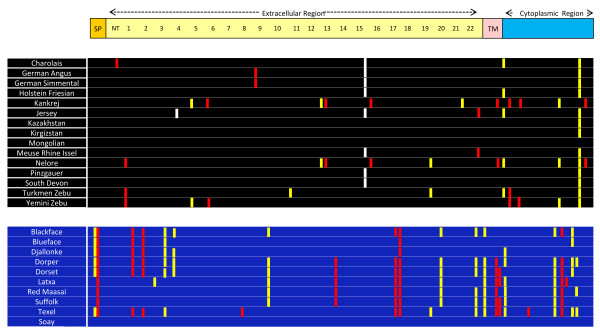
**SNPS detected in TLR5.** SNPs detected for each breed aligned to predicted secondary structure of TLR5. SP = Signal peptide; NT = LRR N-terminal; 1 – 22 in yellow = denotes each LRR; CT = LRR C-terminal; TM = transmembrane region; red bars = Non-synonymous polymorphisms; yellow bars = synonymous polymorphisms; White bars = putative stop codons; Black table = cattle breeds; Blue table = sheep breeds.

A total of 19 polymorphic sites were detected in cattle and 25 in sheep. No overlap between cattle and sheep was seen (Additional file 3 and Additional file 4). All but the Mongolian cattle breed and the Soay sheep breed showed variability within tested individuals (Figure 3).

Of the 19 polymorphisms detected in cattle, 8 are synonymous substitutions and 11 are non-synonymous substitutions. Twelve of the SNPs detected have been previously reported [14,15]. The remaining 7 SNPs were novel discoveries of this study, and three of these were non-synonymous (L34P, R59K and H262R). All except two polymorphisms are sub-species specific, with *Bos indicus* displaying the highest degree of genetic variability (details in supplementary information). Analysis of sheep breeds identified 25 novel SNPs, of which 13 were synonymous and 12 were non synonymous substitutions (Figure 3). In addition, two SNPs were identified which are predicted to cause premature stop codons in cattle TLR5 (Figure 3 and Additional file 4). R125* was detected in the Jersey breed and has recently been reported (15). The other putative stop codon (S431*) was detected in a selection of *Bos taurus* breeds and is a novel discovery of this study. Pseudogenes are predicted to evolve under neutral selection and as such are not subject to the same evolutionary constraints assumed for protein coding genes. Thus to avoid possible problems of including potential pseudogenes in evolutionary analyses, the stop codon variants were excluded from PAML analysis.

### Co-localisation of SNPs and Positively-Selected Sites

**Figure 4:**
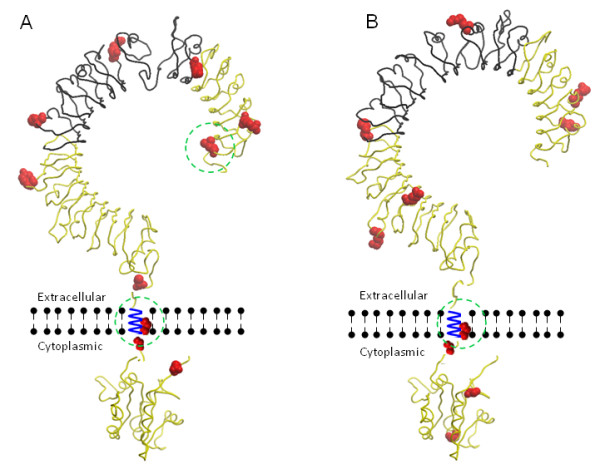
**Localisation of TLR5 SNPs.** Predicted tertiary protein structure of TLR5 and non-synonymous SNPs. **A**) Predicted tertiary structure of bovine TLR5 and positions of non-synonymous SNPs. 10 amino acid sites correspond to 11 detected SNPs as F679L is affected by two different SNPs in of the same codon. Dashed green circles indicate SNPs occurring at sites of positive selection (L34 and A659); **B**) Predicted tertiary structure of ovine TLR5 and positions of non-synonymous SNPs. Black backbone depicts putative flagellin binding region; Red amino acids = Non-synonymous SNPs; Dashed green circle indicate SNP occurring at sites of positive selection (A659 corresponding to M659 in sheep).

Two non-synonymous SNPs detected in the bovine species co-occurred at codons detected as evolving under positive selection. Codon L34 (*d*_*N*_*/d*_*S*_ >1 in artiodactyls) is positioned at the N-terminal region of the extracellular domain of TLR5. Amino acid A659 (*d*_*N*_*/d*_*S*_ >1 in all mammals) is located in the transmembrane region and is predicted to extend this domain (Figure 4 and Additional file 3 and Additional file 5). In sheep, two non-synonymous SNPs at position 2 and 3 of codon A659 were also detected (Figure 4 and Additional file 4). Due to the relatively low density of SNPs in close proximity within a single gene, these were not used to estimate population genetic measures such as linkage disequilibrium.

## Discussion

Whilst many studies of the phylogeny and comparative genomics of the TLR gene family exist, this is the first study to characterize the lineage-specific adaptive evolution of the TLR5 gene across clades of the mammalian phylogeny. Three codons (G104, H592, A659) exhibited clear evidence for positive selection across the mammalian phylogeny. This extends previous discoveries that detected positive selection in TLR5 in primate species [5] or in selected mammalian species [6]. Areal et al. showed that positive selection is seen in a number of genes of the TLR family of proteins including multiple sites of positive selection in the TLR5 genes when using a subset of mammals [6]. Whilst our findings largely support those of their study Areal *et al* used a reduced group of animals to identify positive selection. Seventeen species were studied compared to the 37 species of mammal used in this study. The current study is proposed to have increased power to resolve true signature of positive selection. Importantly the Areal study included the chicken TLR5 gene sequence (NM_001024586.1) in the analysis of TLR5 (see Table S5 of [6]). This would potentially introduce the problem of saturation (see Methods section). Additionally, our study included the novel analysis of groups of lineages to investigate changes in evolutionary constraint in different lineages within the mammals. When *d*_*N*_*/d*_*S*_ between lineages or clades were compared (single branch-sites and multiple branch-sites analysis) the artiodactyl lineage and the individual porcine, ovine and bovine lineages comprising this clade exhibited significant evidence of adaptive evolution (*d*_*N*_*/d*_*S*_ >1). Within the artiodactyl clade, eight positively-selected sites were detected. Positive selective pressure on genes is symptomatic of functional adaptations acquired during the evolution of species and can promote species functional diversification [16]. This suggests that positive selection observed within the artiodactyl clade is different when compared to that seen in other mammals. We postulate that adaptive evolution observed in TLR5 of domestic livestock is a result of the breeding process. In support of this, it has been previously proposed that ruminant species are undergoing differential selective pressure in the related TLR2 genes [17]. This phenomenon may be directly caused by selective breeding resulting in a rapidly restricted population. The effective population size of all cattle breeds is known to have decreased in recent history and this may reflect initial domestication, breed formation or selection for breed specific production traits (beef or milk) [18]. However, the genetic diversity of cattle as opposed to other species such as dogs is not as low as the effective population size would suggest and high levels of divergent selection associated with immune genes amongst others are detectable [18]. As genes such as those of the MHC show balancing selection between breeds [18], an alternative proposal is that the breeding process indirectly drove changes in host-pathogen interactions. By increasing animal density, pathogen transmission and load may also have been increased providing the selective drive for rapid adaptation of host and pathogen genes. Eleven of the 13 positively-selected sites are positioned in the ECD of TLR5 – the part of the protein involved in flagellin recognition. It is known that variability within this region influences species-specific ligand recognition [7]. Evidence of increased positive selection within the extracellular domain supports the hypothesis that competition with flagellated bacterial pathogens is driving adaptation in specific host TLR5 ECD and more precisely in the flagellin-binding region. This may be counterintuitive as many livestock will be expected to share similar microbiota and pathogens. However, TLR5-flagellin interaction has recently been mapped at the single amino acid level [7,19,20] suggesting that changes of amino-acids within either, TLR5 or flagellin can alter the species-specific TLR5 response. This in turn may influence the host range and susceptibility of infection. Such amino-acid changes could explain some of the biological differences seen in the response of different species to flagellin. Indeed, chicken TLR5 has been shown to recognise different flagellin-forms compared to human or murine TLR5 [8], and bovine TLR5 has been shown to have a reduced response to recombinant FliC of *S. typhimurium*[21]. These differences are partially based on flagellin-amino-acid differences, and alteration of these amino acids in different flagellins alters their interaction with TLR5 from different species [8,22]. It has been proposed that PAMP ligands engage with the concave surface of their cognate TLR ECDs [23]. Five codons (S268, I295, I307, K326, I393) under positive selection in the artiodactyl clade are in close proximity to this conserved cavity. The non-synonymous SNPs detected within the putative flagellin-binding region are good candidates for genetic variants most likely to impact upon the immune response mediated by TLR5. The function of some of these variants is currently being pursued. However, sites more distal to the flagellin-binding region may also be of importance in TLR ligand recognition and function, for example by altering the shape of the molecule or interfering with signal transduction. For example, in TLR3 a SNP outside of the extracellular region was found to impair receptor signalling [24]. Also a polymorphism in the transmembrane region of human TLR1 is found to regulate the innate immune response indicating that the transmembrane region plays a role in function [25].

Site-specific co-incidence of adaptive codons (*d*_*N*_*/d*_*S*_ >1) and detection of SNPs suggests that adaptive evolution within these regions is on-going. A good example is codon A659, which is identified as evolving under positive selection across the mammalian phylogeny and also three non synonymous SNPs (2 in sheep, 1 in *Bos indicus*) that were found in this codon. SNPs detected at these positions in both sheep and cattle argues against A659 variation being stochastic but is likely to convey an advantage. A previous study of bovine TLR5 SNPs surmised that this site may be of functional significance (SNPdb ID: rs55617251) [26]. This finding was supported using the SIFT program which predicts the functional relevance of SNPs by comparison of conservation at that site [27]. We used the same approach to confirm this result but found that the SNP is now predicted to be a tolerated replacement. This conflict in results suggests caution when solely relying on algorithmic methods to predict putative sites of functional relevance. However, secondary structure analysis on bovine TLR5 revealed this SNP is predicted to alter the alpha helix structure (Additional file 5). This suggests that this amino acid site may indeed be of functional significance.

As expected, we found the internal signaling TIR domain to have evolved predominantly under purifying selection. In cattle a single codon (H720) was predicted as evolving subject to adaptive evolution. This positively-selected site is close to the BB-loop which is predicted to be involved in TLR dimerization and adaptor protein recognition [28].

Of the 25 breeds investigated in our study two breeds were found to have no detectable SNPs in TLR5. The Soay sheep breed was found to be homozygous in the 10 individuals analysed. The Soay breed is a primitive domestic breed which was introduced to Soay island of the St. Kilda archipelago in the Outer Hebrides of northern Scotland. This breed has experienced expansive population growth followed by seasonal periods of population crashes thought to be associated with parasitic helminth disease [29]. Our results indicate that this breed may have lost heterozygosity within TLR5. This raises the possibility that opportunistic bacterial infections may occur due to a poor TLR5 repertoire in this breed. However, sampling of a larger sample set for this breed should be carried out to verify this result.

Four cattle breeds (Kankrej, Nelore, Turkmen Zebu, Yemini Zebu) included in this study were *Bos indicus* subspecies and were found to contain a higher proportion of the total number of non-synonymous SNPs along with all, except two, of the total number of synonymous SNPs (Additional file 6) suggesting that the SNPs are evolutionarily recent events following the divergence of *Bos indicus* and *Bos taurus* (estimated to have diverged as early as 200,000 years ago) [30].

## Conclusions

We have shown that in agreement with other studies, positive selection is acting on mammalian TLR5. However, our analyses have revealed that the domesticated species in the artiodactyl clade have undergone detectable diversifying selection compared with the rest of the mammals. With the ubiquity of bacterial infection why should the ungulate clade be different to the rest of the mammals? We suggest that artificial selection may have accelerated the evolutionary process resulting in positive selective pressure driving adaptation of the TLR5 gene. The nature of this selection is not known but may be due to selection for cattle resistant to bacteria during domestication or due to an increased bacterial load due to maintaining animals in closer groups and promoting bacterial infection. The concentration of positively-selected sites in close proximity to the conserved cavity of the protein supports the hypothesis that on-going competition is a driving force shaping both the bacterial flagellin and host TLR5 genes.

## Methods

### Identification of positive selection

Genes encoding mammalian TLR5 were obtained from Ensembl and GenBank (Additional file 7). Translated protein sequences were aligned using Muscle [31] and back translated to obtain a codon alignment. Sequences with an in frame stop codon were removed, and 37 sequences remained (Additional file 8). Phylogenetic analysis was based on an accepted mammalian phylogeny [32] from which an unrooted tree of aligned species was created (Additional file 9). Using this tree topology branch lengths were calculated by codeML using the M0 codon model in PAML package version 4 [11]. This tree was used as the fixed tree topology for subsequent analysis. The F3 × 4 codon frequency model calculated using the nucleotide frequencies at the three codon positions was used throughout the analysis. To detect positive selection at individual codons within a gene (sites analysis) two pairs of models were compared using codeML: M1a (neutral model) was compared with M2a (adaptive model) [33,34] and M7 (beta) was compared with M8 (beta plus omega) [35]. Statistically-significant evidence of positive selection was inferred by a likelihood ratio test (LRT) comparing 2 × the log likelihood difference of each set of nested models. These values were compared to the χ^2^ distribution with 2 degrees of freedom. To ensure convergence, the analysis was conducted in triplicate with varying initial *d*_*N*_*/d*_*S*_ values. The assumption that *d*_*N*_*/d*_*S*_ is equal along all lineages of a phylogeny is likely to be false therefore to identify evidence of episodic selection in specific lineages the branch sites test [36] was also used. This approach allows the *d*_*N*_*/d*_S_ values at each codon to vary between an *a priori defined* specific branch (foreground) where adaptive evolution is allowed (*d*_*N*_*/d*_*S*_ >1) and the rest of the tree (background) where adaptive selection is not allowed *d*_*N*_*/d*_*S*_ = 1. Using the branch-sites test, two comparisons were conducted: (1) each branch in turn was analysed as the foreground branch and compared with the rest of the tree and (2) a multiple branch site analysis was conducted, where major taxonomic groups of animals were each in turn grouped as the foreground (*d*_*N*_*/d*_*S*_ > 1) lineage and the remaining lineages are grouped as background (*d*_*N*_*/d*_*S*_ = 1). Clades compared were primates, artiodactyls, laurasiatheria and euarchontoglires (Figure 1). This approach of comparison of multiple branches has been shown to improve the power of the branch-sites analysis if the underlying biological assumption to group the branches is sound [37]. This approach directly tests adaptive evolution in multiple branches and avoids the assumptions of the clades analysis model (CmC [38]) which has been recently criticized [39]. In additional the branch-sites test has been criticized as being prone to false positives when strong positive selection exists in the background branches [40]. To account for this reciprocal experiments are conducted where foreground and background branches are reversed and retested. None of these reciprocal experiments resulted in significant results, suggesting that strong positive selection of background branches does not account for the positive selection seen in foreground branches. In all branch-sites tests the Bonferroni correction [41] was employed to control the type I error rate when comparing multiple foreground lineages. In all tests if the LRT identified that a model allowing positive selection significantly improved the likelihood of the data (P < 0.05 using Bonferroni corrected χ^2^ critical values) codons subject to adaptive evolution were then inferred using the Bayes empirical Bayes algorithm [42]. Codons with a posterior probability of > 95% (P_*b*_ > 0.95) of belonging to the class *d*_*N*_*/d*_*S*_ >1 are reported as having been subject to positive selection. All positions reported correspond to bovine TLR5 (accession: NP_001035591.1). There has been recent discussion of the appropriateness of the branch-sites method to detect positive selection particularly by Nozawa et al. [43] who suggested that the branch-sites test is particularly susceptible to false positives when the number of nucleotide substitutions is small. Nozawa et al. proposed that a minimum of 9 substitutions are required to accurately infer positive selection along a foreground branch [43]. Whilst the validity of the approach used to reach these conclusions has also been challenged [44,45], the data analyzed here does fulfill Nozawa’s suggestions. Caution should also be exercised when the number of substitutions along a lineage is too great and can lead to errors in predictions. This is due to the problem of saturation of substitutions where the true number of substitutions is masked by multiple nucleotide changes at a single site. The branches of the species of interest are not considered to be a concern in this regard. For example, the combined branch lengths of the artiodactyl clade (cow, sheep and pig) are less than one substitution per codon. Whilst the results of any inference should be subject to future validation we are confident of the appropriateness of the tests used.

### Protein Structure Prediction

The domain architecture for TLR5 was determined by LRR finder [46], SMART6 [47] and TMHMM [48]. This largely follows published predictions [3]. Secondary Structure predictions were completed using PSI PRED [49]. Tertiary structure predictions were generated using Swiss-Model [50] using known crystallised TLR structures of highest similarity: extracellular domain template 2a0zA and TIR domain template 1fyvA. Positions of sites of positive selection and SNPs affecting particular amino acid positions were visualised using MolSoft ICM Browser [51].

### Sequence Assembly and SNP Detection

Bovine genomic DNA (n = 110) representing 15 breeds (Additional file 10), and ovine genomic DNA (n = 87) representing 10 breeds (Additional file 11), were extracted from blood samples using standard procedures (Qiagen, UK)*.* Samples were not obtained for the sole purpose of this project, but were either donated (see acknowledgements) or from existing DNA banks at the Roslin Institute. The single exon encoding TLR5 was amplified using primers designed using the bovine sequence (Btau 4.0) (Additional file 12 Table S6 and Additional file 13). Amplification was performed using Taq polymerase (cattle) and KOD proof reading enzyme (sheep). Sequence reads were generated using 6 sequencing primer sets for cattle and 4 for sheep using BigDye Terminator (Applied Biosystems).

Sequences were assembled for each breed using the programs Pregap and Gap4 from the Staden sequence analysis package [52]. Variations for individuals and between breeds were detected using Contig editor. SNPs were included in analysis only when polymorphisms were detected at sites of high confidence (*Phred* score >30). Consensus sequences for TLR5 from each breed were exported using Gap4 and polymorphic sites reported by MEGA4 [53]. Nucleotide sequences were translated to amino acid sequences to ascertain the impact of each SNP.

## Competing interests

The authors declare that they have no competing interests.

## Authors' contributions

SAS designed and conducted the molecular genetic studies, performed the statistical analysis and drafted the manuscript. OCJ designed amplification primers for TLR5 and facilitated amplification and sequencing of cattle samples. OCJ, DH, GCR, DW and EJG participated in the design of the study. DH and EJG conceptualised and coordinated the study. RDE participated in conceptualisation and study design, performed the statistical analysis and helped to draft the manuscript. All authors have read and approved the final manuscript.

## Supplementary Material

Additional file 1 Results and parameter estimates of all single branch-sites tests. Click here for file

Additional file 2 Results and parameter estimates of all multiple branch-site analysis. Click here for file

Additional file 3 **Positions of detected bovine SNPs.** Results of SNP detection across 15 breeds of cattle. W/T refers to wildtype; Mut refers to mutation form. NS = non-synonymous polymorphism; S = synonymous polymorphism. Amino acid one letter code names amino acid by convention. The type of nucleotide for each SNP and each breed is recorded using IUBMB single-letter code for nucleotide bases and ambiguity codes: R = A/G; Y = C/T; M = A/C; W = A/T; S = C/G; K = G/T. Click here for file

Additional file 4 **Positions of detected ovine SNPs.** Results of SNP detection across 10 breeds of sheep. W/T refers to wildtype; Mut refers to mutation form. NS = non-synonymous polymorphism; S = synonymous polymorphism. Amino acid one letter code names amino acid by convention. The type of nucleotide for each SNP and each breed is recorded using IUBMB single-letter code for nucleotide bases and ambiguity codes: R = A/G; Y = C/T; M = A/C; W = A/T; S = C/G; K = G/T. Click here for file

Additional file 5 Secondary structure sequence predictions (PSI-pred) affecting SNP A659T in cattle TLR5. Click here for file

Additional file 6 **Subspecies distribution of SNPs detected in Bovine TLR5.** S: = synonymous SNP; NS: = non-synonymous SNP. Click here for file

Additional file 7 **Accession numbers of all sequences compared.** Accession numbers of TLR5 coding nucleotide sequences used for PAML analysis. Click here for file

Additional file 8 Alignment of all TLR5 genes analysed in Fasta format. Click here for file

Additional file 9 Phylogenetic tree of all TLR5 genes analysed. Click here for file

Additional file 10 **Details of bovine DNA samples.** Bovine DNA samples. Sample size and subspecies characterization for each breed is detailed. Click here for file

Additional file 11 **Details of ovine DNA samples.** Ovine DNA sample set. Sample size for each breed is detailed. Click here for file

Additional file 12 **Bovine primer sequences Primers used for the sequencing of the coding sequence of bovine TLR5.** Forward primer 1 and Reverse Primer 6 are positioned in the un-translated regions either side of the single exon of TLR5. Click here for file

Additional file 13 **Ovine primer sequences.** Ovine TLR5 sequencing primers. Forward primer 1 and reverse primer 4 are positioned in the un-translated region either side of the single exon of TLR5. Click here for file
