# Supplementary material for: Adaptive evolution of Toll-like receptor 5 in domesticated mammals
Source: BMC Evol Biol. 2012 Jul 24;12:122. doi: 10.1186/1471-2148-12-122 (PMC3483281; doi:10.1186/1471-2148-12-122)
Supplement: Additional file 1 — Results and parameter estimates of all single branch-sites tests. [file 1471-2148-12-122-S1.doc]

Significant Foreground Branch Log likelihood fixed Log likelihood free 2x (InL free-InL fixed) tree length fixed tree length free kappa fixed kappa free p0 fixed p0 free p1 fixed p1 free p2 fixed p2 free Bg-w fixed Bg-w free Fg-w fixed Fg-w free foreground branch length (substitutions/codon)

0.001 Cow -24692.263 -24681.343 21.84 8.58926 8.78344 3.362 3.377 0.568 0.648 0.299 0.339 0.133 0.013 0.092 0.093 1 140.906 0.121331

0.01 Sheep -24697.761 -24688.712 18.098 8.58542 8.98439 3.36 3.377 0.62 0.648 0.325 0.34 0.055 0.012 0.094 0.094 1 493.283 0.085169

0.05 Sloth -24694.893 -24688.931 11.924 8.58732 8.63654 3.365 3.379 0.6 0.643 0.315 0.333 0.085 0.025 0.093 0.094 1 12.673 0.249991

0.05 branch to pig -24694.65 -24688.706 11.888 8.58956 8.83227 3.365 3.387 0.621 0.644 0.326 0.337 0.054 0.019 0.092 0.093 1 29.068 0.459224

Hedgehog -24698.079 -24697.537 1.084 8.57908 8.59801 3.361 3.365 0.646 0.648 0.34 0.341 0.014 0.01 0.094 0.094 1 3.237 1.116724

Cat -24698.537 -24698.537 0 8.58121 8.5812 3.361 3.361 0.655 0.655 0.345 0.345 0 0 0.095 0.095 1 1 0.296005

Dog -24694.847 -24692.57 4.554 8.58214 9.11205 3.36 3.375 0.621 0.644 0.327 0.339 0.052 0.017 0.091 0.093 1 31.275 0.853459

Giant_Panda -24698.537 -24698.537 0 8.58121 8.5812 3.361 3.361 0.655 0.655 0.345 0.345 0 0 0.095 0.095 1 1 0.306801

Hyrax -24697.925 -24697.764 0.322 8.58121 8.58426 3.361 3.363 0.637 0.649 0.335 0.342 0.028 0.009 0.094 0.094 1 3.315 0.413152

Tenrec -24698.334 -24693.321 10.026 8.58193 9.25718 3.361 3.378 0.646 0.649 0.34 0.338 0.014 0.013 0.094 0.094 1 86.779 0.496915

Pika -24698.518 -24698.518 0 8.58194 8.58193 3.361 3.361 0.651 0.651 0.342 0.342 0.007 0.007 0.094 0.094 1 1 0.379026

Rabbit -24698.305 -24696.947 2.716 8.58205 8.59189 3.362 3.366 0.648 0.652 0.342 0.345 0.011 0.003 0.094 0.094 1 15.061 0.288449

Pig Sscrof1 -24698.537 -24698.537 0 8.58118 8.58118 3.361 3.361 0.655 0.655 0.345 0.345 0 0 0.095 0.095 1 1 0.005052

Pig BLWx -24698.497 -24698.497 0 8.58116 8.5812 3.361 3.361 0.628 0.628 0.331 0.331 0.041 0.041 0.095 0.095 1 1 0.011749

Rat -24698.534 -24698.534 0 8.58153 8.58155 3.361 3.361 0.653 0.653 0.344 0.344 0.003 0.003 0.095 0.095 1 1 0.210504

Mouse 1 -24696.468 -24695.979 0.978 8.58334 8.58313 3.361 3.361 0 0 0 0 1 1 0.095 0.095 1 153.712 0.002846

Mouse 2 -24698.537 -24698.537 0 8.58122 8.58121 3.361 3.361 0.655 0.655 0.345 0.345 0 0 0.095 0.095 1 1 0.000694

Homo sapiens -24697.031 -24697.031 0 8.58234 8.58233 3.36 3.36 0.362 0.362 0.191 0.191 0.447 0.447 0.094 0.094 1 1 0.010439

Pongo_pygmaeus_gi_222788848 -24698.537 -24698.537 0 8.58114 8.5812 3.361 3.361 0.655 0.655 0.345 0.345 0 0 0.095 0.095 1 1 0.001187

Pongo_pygmaeus_gi_194068450 -24698.537 -24698.537 0 8.58121 8.58121 3.361 3.361 0.655 0.655 0.345 0.345 0 0 0.095 0.095 1 1 0.001163

Gorilla_gorilla_gi_222788846 -24698.145 -24698.13 0.03 8.58131 8.58149 3.362 3.362 0.514 0.605 0.271 0.319 0.215 0.076 0.094 0.094 1 2.99 0.005887

Pan_troglodytes_gi_222790154 -24697.276 -24697.276 0 8.58155 8.58136 3.362 3.362 0 0 0 0 1 1 0.094 0.094 1 1 0.002359

9 -24698.537 -24698.537 0 8.58124 8.5812 3.361 3.361 0.655 0.655 0.345 0.345 0 0 0.095 0.095 1 1 0.001262

8 -24698.517 -24698.492 0.05 8.58131 8.5813 3.361 3.361 0.638 0.647 0.336 0.341 0.026 0.012 0.095 0.095 1 3.673 0.013162

3 -24698.537 -24698.537 0 8.58121 8.58122 3.361 3.361 0.655 0.655 0.345 0.345 0 0 0.095 0.095 1 1 0.155168

12 -24698.537 -24698.537 0 8.58122 8.58123 3.361 3.361 0.655 0.655 0.345 0.345 0 0 0.095 0.095 1 1 0.007993

13 -24695.463 -24695.463 0 8.58263 8.58268 3.363 3.363 0.151 0.151 0.08 0.08 0.769 0.77 0.094 0.094 1 1 0.010771

16 -24698.446 -24698.446 0 8.58151 8.58149 3.361 3.361 0.627 0.627 0.33 0.33 0.042 0.042 0.094 0.094 1 1 0.028462

17 -24695.837 -24695.837 0 8.58216 8.58213 3.364 3.364 0.203 0.203 0.108 0.108 0.69 0.689 0.094 0.094 1 1 0.010203

20 -24698.306 -24698.302 0.008 8.58173 8.58176 3.362 3.362 0.612 0.623 0.323 0.329 0.065 0.048 0.094 0.094 1 1.378 0.029266

21 -24698.537 -24698.537 0 8.58124 8.58121 3.361 3.361 0.655 0.655 0.345 0.345 0 0 0.095 0.095 1 1 0.012294

33 -24698.537 -24698.537 0 8.58121 8.58123 3.361 3.361 0.655 0.655 0.345 0.345 0 0 0.095 0.095 1 1 0.043775

34 -24695.571 -24693.174 4.794 8.58698 8.5924 3.364 3.366 0.619 0.646 0.328 0.343 0.053 0.012 0.093 0.092 1 7.87 0.219343

37 -24698.006 -24698.006 0 8.58367 8.58369 3.363 3.363 0.622 0.622 0.328 0.328 0.051 0.051 0.094 0.094 1 1 0.171969

39 -24694.987 -24693.038 3.898 8.5881 8.60148 3.359 3.363 0.626 0.642 0.327 0.335 0.046 0.023 0.092 0.093 1 3.584 0.549641

42 -24698.537 -24698.537 0 8.58117 8.5812 3.361 3.361 0.655 0.655 0.345 0.345 0 0 0.095 0.095 1 1 0.220385

43 -24697.919 -24695.08 5.678 8.58256 8.59894 3.361 3.364 0.634 0.65 0.335 0.344 0.031 0.006 0.094 0.094 1 66.734 0.082989

44 -24698.537 -24698.537 0 8.58119 8.58121 3.361 3.361 0.655 0.655 0.345 0.345 0 0 0.095 0.095 1 1 0.044795

51 -24698.537 -24698.537 0 8.58118 8.58122 3.361 3.361 0.655 0.655 0.345 0.345 0 0 0.095 0.095 1 1 0.251621

55 -24698.537 -24698.537 0 8.5812 8.5812 3.361 3.361 0.655 0.655 0.345 0.345 0 0 0.095 0.095 1 1 0.289173

56 -24698.701 -24698.537 0.328 8.58575 8.58125 3.361 3.361 0.522 0.655 0.275 0.345 0.203 0 0.095 0.095 1 1 0.018403

57 -24698.283 -24698.283 0 8.58216 8.58217 3.361 3.361 0.386 0.406 0.204 0.214 0.41 0.381 0.094 0.094 1 1 0.008434

59 -24698.537 -24698.537 0 8.5812 8.5812 3.361 3.361 0.655 0.655 0.345 0.345 0 0 0.095 0.095 1 1 0.058925

60 -24698.465 -24694.647 7.636 8.58104 8.60623 3.36 3.366 0.64 0.654 0.336 0.343 0.025 0.003 0.095 0.094 1 203.754 0.04816
