# Supplementary material for: Adaptive evolution of Toll-like receptor 5 in domesticated mammals
Source: BMC Evol Biol. 2012 Jul 24;12:122. doi: 10.1186/1471-2148-12-122 (PMC3483281; doi:10.1186/1471-2148-12-122)
Supplement: Additional file 2 — Results and parameter estimates of all multiple branch-site analysis. [file 1471-2148-12-122-S2.doc]

Foreground euarchontoglires InL tree_len kappa p0 p1 p2 bg-w fg-w

fixed -24697.519 8.59685 3.364 0.643 0.333 0.024 0.092 1

free -24697.519 8.59686 3.364 0.643 0.333 0.024 0.092 1

Foreground NOT euarchontoglires InL tree_len kappa p0 p1 p2 bg-w fg-w

fixed -24689.569 8.59308 3.349 0.642 0.298 0.06 0.09 1

free -24689.569 8.59312 3.349 0.642 0.298 0.06 0.09 1

----------------------------------------------------------------------------------------------------

Foreground laurasiatheria InL tree_len kappa p0 p1 p2 bg-w fg-w

fixed -24684.329 8.59773 3.346 0.635 0.304 0.061 0.088 1

free -24684.329 8.59777 3.346 0.635 0.304 0.061 0.088 1

Foreground NOT laurasiatheria InL tree_len kappa p0 p1 p2 bg-w fg-w

fixed -24691.331 8.6133 3.367 0.639 0.321 0.04 0.089 1

free -24691.331 8.61331 3.367 0.639 0.321 0.04 0.089 1

----------------------------------------------------------------------------------------------------

Foreground artiodactyla InL tree_len kappa p0 p1 p2 bg-w fg-w

fixed -24678.459 8.62035 3.363 0.606 0.31 0.084 0.088 1

free -24660.655 8.71386 3.424 0.625 0.316 0.059 0.091 4.759

Sites: 34L* 269S** 296I* 308I* 394I* 499Q* 625I* 634E**

Foreground NOT artiodactyla InL tree_len kappa p0 p1 p2 bg-w fg-w

fixed -24683.675 8.58626 3.341 0.641 0.21 0.149 0.088 1

free -24683.675 8.58625 3.341 0.641 0.21 0.149 0.088 1
