# Supplementary figures and images for: Adaptive evolution of Toll-like receptor 5 in domesticated mammals
Source: BMC Evol Biol. 2012 Jul 24;12:122. doi: 10.1186/1471-2148-12-122 (PMC3483281; doi:10.1186/1471-2148-12-122)

**
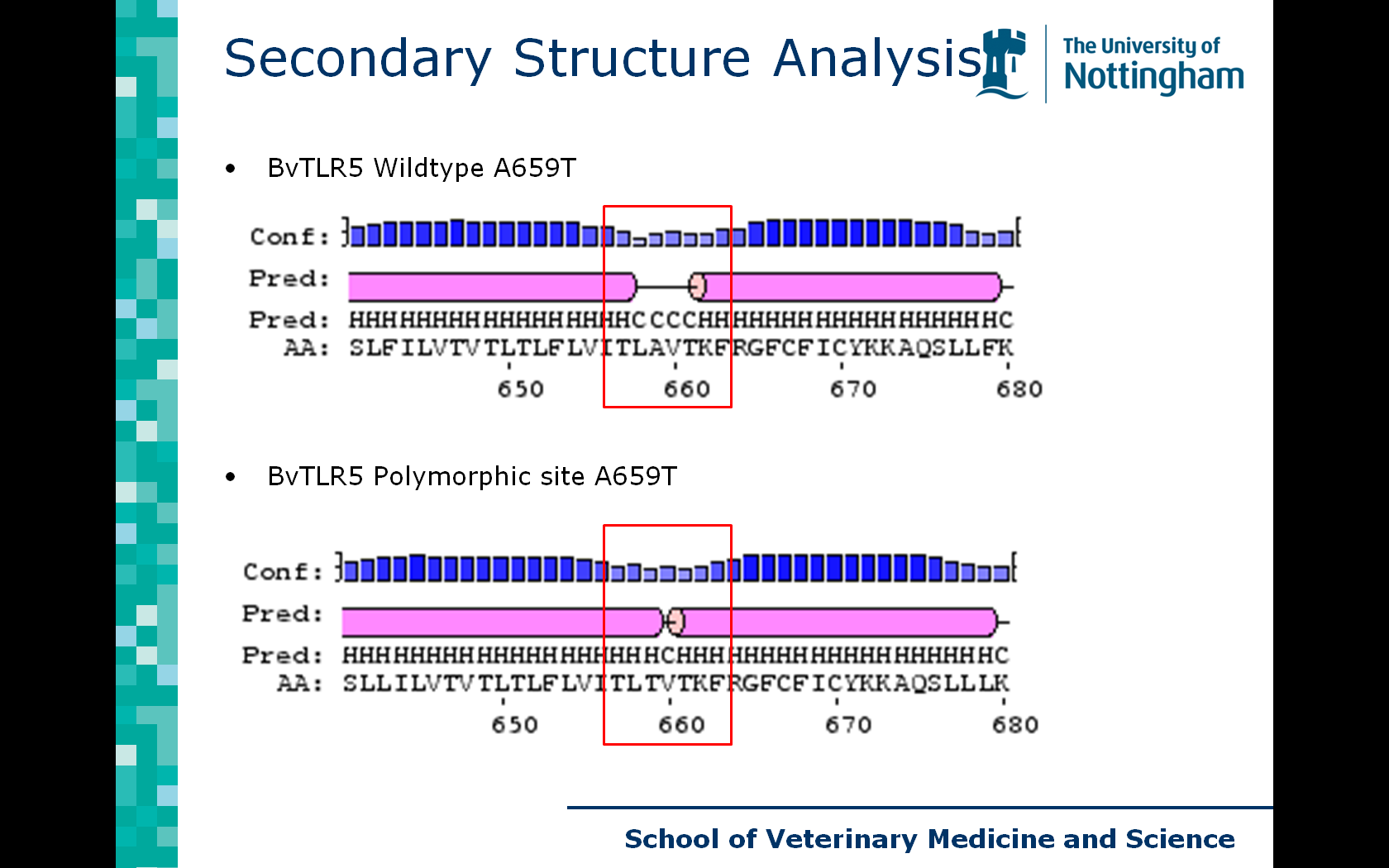
**

Supplement: Additional file 5 — Secondary structure sequence predictions (PSI-pred) affecting SNP A659T in cattle TLR5. [file 1471-2148-12-122-S5.doc]
