# Supplementary material for: Adaptive evolution of Toll-like receptor 5 in domesticated mammals
Source: BMC Evol Biol. 2012 Jul 24;12:122. doi: 10.1186/1471-2148-12-122 (PMC3483281; doi:10.1186/1471-2148-12-122)
Supplement: Additional file 7 — Accession numbers of all sequences compared. Accession numbers of TLR5 coding nucleotide sequences used for PAML analysis. [file 1471-2148-12-122-S7.doc]

Mammalian species used for PAML analysis

| **Common name** | **Latin name** | **Databank** | **Accession Number** |
| --- | --- | --- | --- |
| Hedgehog | *Erinaceus europaeus* | Ensembl | ENSEEUT00000003134 |
| Sloth | *Choloepus hoffmanni* | Ensembl | ENSCHOT00000011335 |
| Cat | *Felis catus* | Ensembl | ENSFCAT00000006837 |
| Dog | *Canis familiaris* | Ensembl | ENSCAFT00000018059 |
| Giant Panda | *Ailuropoda melanoleuca* | Ensembl | ENSAMET00000022014 |
| Hyrax | *Procavia capensis* | Ensembl | ENSPCAT00000009659 |
| Tenrec | *Echinops telfairi* | Ensembl | ENSETET00000013405 |
| Pika | *Ochotona princeps* | Ensembl | ENSOPRT00000013962 |
| Rabbit | *Oryctolagus cuniculus* | Ensembl | ENSOCUT00000025682 |
| Cow | *Bos Taurus* | GenBank | NM_001040501.1 |
| Pig | *Sus scrofa* | GenBank | 224384245 |
| Pig | *Sus scrofa* | GenBank | 294719805 |
| Sheep | *Ovis aries* | GenBank | 209693401 |
| Rat | *Rattus norvegicus* | GenBank | 224612142 |
| Mouse | *Mus musculus* | GenBank | 7648686 |
| Mouse | *Mus musculus* | GenBank | 115545512 |
| Human | *Homo sapien* | GenBank | 13810567 |
| Bornean Orangutan | *Pongo pygmaeus* | GenBank | 222788848 |
| Bornean Orangutan | *Pongo pygmaeus* | GenBank | 194068450 |
| Lowland Gorilla | *Gorilla gorilla* | GenBank | 222788846 |
| Common Chimpanzee | *Pan troglodytes* | GenBank | 222790154 |
| Lowland Gorilla | *Gorilla gorilla* | GenBank | 194068448 |
| Black Flying Fox | *Pteropus alectogi* | GenBank | 308055167 |
| Drill | *Mandrillus leucophaeus* | GenBank | 222788864 |
| Olive Baboon | *Papio Anubis* | GenBank | 222788862 |
| Gelada Baboon | *Theropithecus gelada* | GenBank | 222788860 |
| Allen's Swamp Monkey | *Allenopithecus nigroviridis* | GenBank | 222788858 |
| Mona Monkey | *Cercopithecus mona* | GenBank | 222788856 |
| Eastern Black-and-white Colobus | *Colobus guereza* | GenBank | 222788854 |
| Pileated Gibbon | *Hylobates pileatus* | GenBank | 222788852 |
| Siamang | *Symphalangus syndactylus* | GenBank | 222788850 |
| Gracile Chimpanzee | *Pan paniscus* | GenBank | 194068446 |
| Agile Mangabey | *Cercocebus agilis* | GenBank | 222788868 |
| Sooty Mangabey | *Cercocebus torquatus atys* | GenBank | 164652845 |
| Barbary Macaque | *Macaca sylvanus* | GenBank | 222788866 |
| Rhesus Monkey | *Macaca mulatta* | GenBank | 194363738 |
| Crab-eating Macaque | *Macaca fascicularis* | GenBank | 194068452 |
