# Supplementary material for: Adaptive evolution of Toll-like receptor 5 in domesticated mammals
Source: BMC Evol Biol. 2012 Jul 24;12:122. doi: 10.1186/1471-2148-12-122 (PMC3483281; doi:10.1186/1471-2148-12-122)
Supplement: Additional file 10 — Details of bovine DNA samples. Bovine DNA samples. Sample size and subspecies characterization for each breed is detailed. [file 1471-2148-12-122-S10.doc]

| **Breed** | **Sub-species** | **Sample Size TLR5** |
| --- | --- | --- |
| Kankrej | *Bos indicus* | 2 |
| Nelore | *Bos indicus* | 5 |
| Turkmen Zebu | *Bos indicus* | 7 |
| Yemini Zebu | *Bos indicus* | 5 |
| Charolais | *Bos taurus* | 12 |
| German Angus | *Bos taurus* | 11 |
| German Simmental | *Bos taurus* | 10 |
| Holstein Friesian | *Bos taurus* | 7 |
| Jersey | *Bos taurus* | 9 |
| Meuse Rhine Issel | *Bos taurus* | 6 |
| South Devon | *Bos taurus* | 10 |
| Kazakzkaya | Hybrid | 6 |
| Kazakzkaya | Hybrid | 5 |
| Mongolian | Bos taurus | 2 |
| Pinzgauer | *Bos taurus* | 13 |
