# Supplementary material for: Adaptive evolution of Toll-like receptor 5 in domesticated mammals
Source: BMC Evol Biol. 2012 Jul 24;12:122. doi: 10.1186/1471-2148-12-122 (PMC3483281; doi:10.1186/1471-2148-12-122)
Supplement: Additional file 11 — Details of ovine DNA samples. Ovine DNA sample set. Sample size for each breed is detailed. [file 1471-2148-12-122-S11.doc]

| **Breed** | **Source** | **Sample Size TLR5** |
| --- | --- | --- |
| Blueface | MRI | 7 |
| Blackface | MRI | 10 |
| Texel | MRI | 8 |
| Red Maasai | ILRI | 10 |
| Soay | MRI | 10 |
| Suffolk | MRI | 6 |
| Dorset | MRI | 10 |
| Dorper | ILRI | 10 |
| Djallonke | ILRI | 6 |
| Latxa | Neiker | 10 |
