# Supplementary material for: Adaptive evolution of Toll-like receptor 5 in domesticated mammals
Source: BMC Evol Biol. 2012 Jul 24;12:122. doi: 10.1186/1471-2148-12-122 (PMC3483281; doi:10.1186/1471-2148-12-122)
Supplement: Additional file 12 — Bovine primer sequences Primers used for the sequencing of the coding sequence of bovine TLR5. Forward primer 1 and Reverse Primer 6 are positioned in the un-translated regions either side of the single exon of TLR5. [file 1471-2148-12-122-S12.doc]

Cattle TLR5 Sequence Primers

| **Sequence Primer** | **Primer Type** | **Direction** | **Sequence** |
| --- | --- | --- | --- |
| TLR5 Primer Set 1 | Forward | 5’ > 3’ | GCTCAGTGCCTTGAGCTTAGA |
|  | Reverse | 5’ > 3’ | TCAAGGAATTCAGTTCCCG |
| TLR5 Primer Set 2 | Forward | 5’ > 3’ | CCGATGCTGTATTAAAAGATGG |
|  | Reverse | 5’ > 3’ | TTCAGCTCCTGGAGTGTCTC |
| TLR5 Primer Set 3 | Forward | 5’ > 3’ | CCAGGAGCTCGATGATACAG |
|  | Reverse | 5’ > 3’ | GGGCATGGTTTTGGTGAC |
| TLR5 Primer Set 4 | Forward | 5’ > 3’ | TTCCTTCTCCAGGTACCTCATC |
|  | Reverse | 5’ > 3’ | AAAGACTGTAAATGGAAACCCC |
| TLR5 Primer Set 5 | Forward | 5’ > 3’ | ATCACAATAGCTGGGTCTCCA |
|  | Reverse | 5’ > 3’ | CAGGCCACCTCAAGTACTGC |
| TLR5 Primer Set 6 | Forward | 5’ > 3’ | CCCAGAGTCTGCTGTTCAAG |
|  | Reverse | 5’ > 3’ | GGCTTGCGATAAGTGGAAAC |
