# Supplementary material for: Adaptive evolution of Toll-like receptor 5 in domesticated mammals
Source: BMC Evol Biol. 2012 Jul 24;12:122. doi: 10.1186/1471-2148-12-122 (PMC3483281; doi:10.1186/1471-2148-12-122)
Supplement: Additional file 13 — Ovine primer sequences. Ovine TLR5 sequencing primers. Forward primer 1 and reverse primer 4 are positioned in the un-translated region either side of the single exon of TLR5. [file 1471-2148-12-122-S13.doc]

Ovine TLR5 Sequence Primers

| **Sequence Primer** | **Primer Type** | **Direction** | **Sequence** |
| --- | --- | --- | --- |
| Primer Set 1 | Forward | 5’ > 3’ | GCT CAG TGC CTT GAG CTT AGA |
|  | Reverse | 5’ > 3’ | TAG GGT TTC CAG GAC CAT GT |
| Primer Set 2 | Forward | 5’ > 3’ | TGT GAG CAG GAG TTC AAA CC |
|  | Reverse | 5’ > 3’ | AGT GGG ATG TTT GGC AAA GT |
| Primer Set 3 | Forward | 5’ > 3’ | CAT TCA AAT TCC TGG GGA AA |
|  | Reverse | 5’ > 3’ | TCT TCA CAA CCT TCT GTG GAA A |
| Primer Set 4 | Forward | 5’ > 3’ | CAA TAT CAC AAT ATC TGG GTC TCC |
|  | Reverse | 5’ > 3’ | GGC TTG CGA TAA GTG GAA AC |
