# Supplementary material for: Adaptive evolution of Toll-like receptor 5 in domesticated mammals
Source: BMC Evol Biol. 2012 Jul 24;12:122. doi: 10.1186/1471-2148-12-122 (PMC3483281; doi:10.1186/1471-2148-12-122)
Supplement: Supplementary file 11 [file 1471-2148-12-122-S3.doc]

Nucleotide Position	100	176	373	504	541	785	1005	1131	1132	1292	1324	1632	1761	1767	1929	1975	2035	2037	2208	2460	2524
Amino Acid Position	34	59	125	168	181	262	335	377	378	431	442	544	587	589	643	659	679	679	736	820	842
Domain	LRRNT	LRR 1	LRR 4	LRR 5	LRR 6	LRR 9	LRR 11	LRR 13	LRR 13	LRR 16	LRR 16	LRR 20	LRR 22	LRR 22	LRR CT	TRNS	CYTO	CYTO	TIR	TIR	CYTO
Codon W/T	CCT	AGG	CGA	GAA	AAA	CAT	CTC	GAC	AAA	TCA	TAC	GGT	CTT	GCT	TTC	GCG	TTC	TTC	CCG	GAG	GAA
Codon Mut	CTT	AAG	TGA	GAG	GAA	CGT	CTT	GAT	GAA	TGA	CAC	GGC	CTC	GCC	TTG	ACG	CTC	TTG	CCC	GAA	AAA
Amino Acid W/T	L	R	R	E	K	H	L	D	K	S	Y	G	L	A	F	A	F	F	P	E	E
Amino Acid Mut	P	K	Stop	E	E	R	L	D	E	Stop	H	G	L	A	L	T	L	L	P	E	K
SNP Type	NS	NS	NS	S	NS	NS	S	S	NS	NS	NS	S	S	S	NS	NS	NS	NS	S	S	NS
Charolais	Y	G	C	A	A	G	C	C	A	S	T	C	Y	T	C	G	T	C	G	R	G
German Angus	C	G	C	A	A	R	C	C	A	S	T	C	Y	T	C	G	T	C	G	R	G
German Simmental	C	G	C	A	A	R	C	C	A	S	T	C	Y	T	C	G	T	C	G	R	G
Holstein Friesian	C	G	C	A	A	G	C	C	A	S	T	C	Y	T	C	G	T	C	G	R	G
Kankrej	C	G	C	G	G	G	C	Y	R	C	Y	C	T	Y	C	R	Y	S	C	G	A
Jersey	C	G	Y	A	A	G	C	C	A	S	T	C	Y	T	S	G	T	C	G	R	G
Kazakhstan	C	G	C	A	A	G	C	C	A	C	T	C	Y	T	C	G	T	C	G	R	G
Kirgizstan	C	G	C	A	A	G	C	C	A	C	T	C	T	T	C	G	T	C	G	R	G
Mongolian	C	G	C	A	A	G	C	C	A	C	T	C	T	T	C	G	T	C	G	G	G
Meuse Rhine Issel	C	G	C	A	A	G	C	C	A	S	T	C	Y	T	S	G	T	C	G	R	G
Nelore	C	R	C	A	A	G	C	Y	R	C	Y	T	Y	T	C	R	T	C	R	R	R
Pinzgauer	C	G	C	A	A	G	C	C	A	S	T	C	Y	T	C	G	T	C	G	R	G
South Devon	C	G	C	A	A	G	C	C	A	S	T	C	Y	T	C	G	T	C	G	R	G
Turkmen Zebu	C	R	C	A	A	G	Y	C	A	C	T	Y	Y	T	C	G	T	C	G	R	G
Yemini Zebu	C	R	C	A	R	G	C	C	A	C	T	C	Y	T	C	G	T	S	R	A	G
