# Supplementary material for: Adaptive evolution of Toll-like receptor 5 in domesticated mammals
Source: BMC Evol Biol. 2012 Jul 24;12:122. doi: 10.1186/1471-2148-12-122 (PMC3483281; doi:10.1186/1471-2148-12-122)
Supplement: Supplementary file 12 [file 1471-2148-12-122-S4.doc]

**Nucleotide Position**	**33**	**46**	**210**	**275**	**339**	**360**	**396**	**731**	**906**	**1200**	**1474**	**1531**	**1536**	**1698**	**1887**	**1953**	**1976**	**1977**	**2016**	**2047**	**2157**	**2276**	**2353**	**2394**	**2418**
Amino Acid Position	11	16	70	92	113	120	132	244	302	400	492	511	512	566	629	651	659	659	672	683	719	759	785	798	806
Domain	SP	SP	LRR 1	LRR 2	LRR 3	LRR 3	LRR 4	LRR 8	LRR 10	LRR 14	LRR 18	LRR 18	LRR 19	LRR 21	LRR CT	TRNS	TRNS	TRNS	CYTO	CYTO	TIR	TIR	TIR	TIR	TIR
Codon Wildtype	GTC	CGT	GAG	CGA	GAC	CAC	TCC	AGA	TCC	CAT	AAG	CAA	AAC	GAT	TGT	ACT	ATG	ATG	AAG	ATC	TAT	AGC	GGC	TTC	ATC
Codon Mutant	GTT	AGT	GAC	CAA	GAT	CAT	TCT	AAA	TCT	CAG	GAG	AAA	AAT	GAC	TGC	ACC	ACG	ATA	AAA	GTC	TAC	ACC	CGC	TTT	ATT
Amino Acid Wildtype	V	R	E	R	D	H	S	R	S	H	K	Q	N	D	C	T	M	M	K	I	Y	S	G	F	I
Amino Acid Mutant	V	S	D	Q	D	H	S	K	S	Q	E	N	N	D	C	T	T	I	K	V	Y	T	R	F	I
SNP Type	S	NS	NS	NS	S	S	S	NS	S	NS	NS	NS	S	S	S	S	NS	NS	S	NS	S	NS	NS	S	S
Blackface	Y	M	S	R	C	Y	Y	G	Y	T	R	M	C	Y	Y	Y	T	G	G	A	Y	S	G	Y	C
Blueface	Y	M	S	R	C	Y	C	G	C	T	A	M	C	T	T	T	T	G	G	A	T	G	G	Y	C
Djallonke	Y	M	S	R	C	Y	Y	G	C	T	A	M	C	T	T	T	T	G	R	A	T	G	G	C	C
Dorper	Y	M	S	R	C	Y	Y	G	Y	K	R	M	C	Y	Y	Y	Y	R	R	A	Y	S	G	Y	Y
Dorset	Y	M	S	R	C	Y	Y	G	Y	K	R	M	C	Y	Y	Y	Y	R	G	A	Y	S	G	T	C
Latxa	C	M	G	G	Y	C	C	G	Y	K	R	M	C	Y	T	Y	Y	G	R	A	Y	S	S	C	C
Red Maasai	C	M	G	G	C	C	C	G	Y	K	R	M	C	Y	Y	Y	Y	R	R	A	Y	S	G	C	Y
Suffolk	C	M	G	G	C	C	C	G	Y	K	R	M	C	Y	Y	Y	Y	R	R	A	Y	S	G	C	C
Texel	Y	M	S	R	C	Y	C	R	C	T	R	M	Y	T	Y	Y	Y	G	R	R	Y	S	G	Y	Y
Soay	C	C	G	G	C	C	C	G	C	T	A	C	C	T	T	T	T	G	G	A	T	G	G	C	C
