# Supplementary material for: Adaptive evolution of Toll-like receptor 5 in domesticated mammals
Source: BMC Evol Biol. 2012 Jul 24;12:122. doi: 10.1186/1471-2148-12-122 (PMC3483281; doi:10.1186/1471-2148-12-122)
Supplement: Supplementary file 13 [file 1471-2148-12-122-S6.doc]

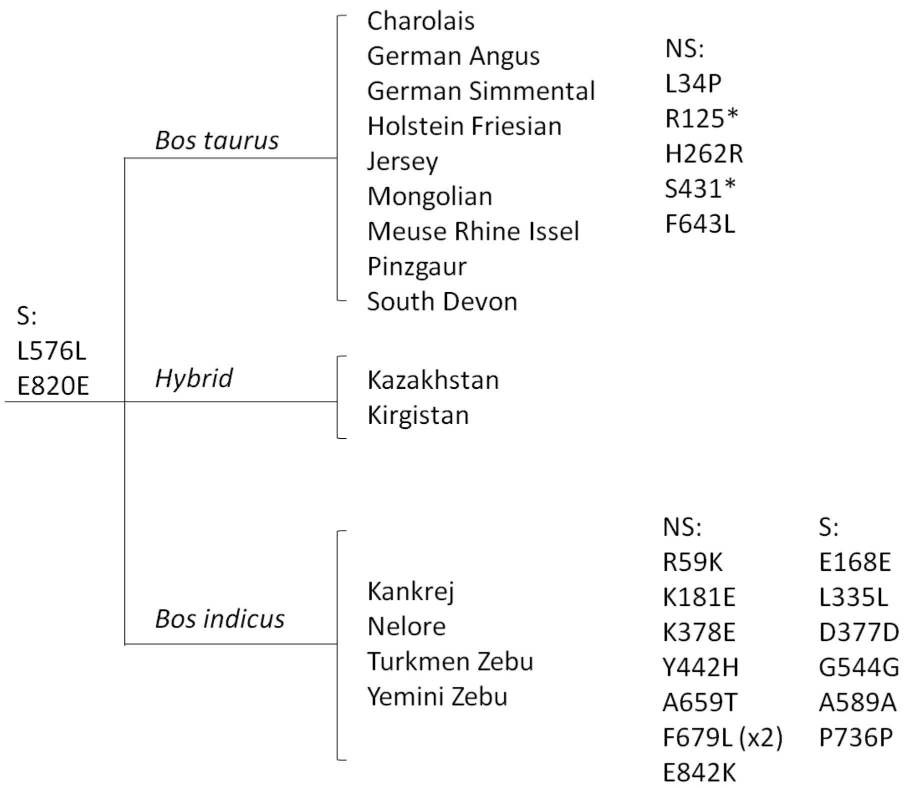
S:
L576L
E820E
Bos taurus
Hybrid
Bos indicus
Charolais
German Angus
German Simmental
Holstein Friesian
Jersey
Mongolian
Meuse Rhine Issel
Pinzgaur
South Devon
NS:
L34P
R125*
H262R
S431*
F643L
Kazakhstan
Kirgistan
Kankrej
Nelore
Turkmen Zebu
Yemini Zebu
NS:
R59K
K181E
K378E
Y442H
A659T
F679L (x2)
E842K
S:
E168E
L335L
D377D
G544G
A589A
P736P
